# Seaweed Extracts to Control Postharvest Phytopathogenic Fungi in Rocha Pear

**DOI:** 10.3390/jof9020269

**Published:** 2023-02-17

**Authors:** Eloísa Toledo, Carina Félix, Tânia F. L. Vicente, Ana Augusto, Rafael Félix, Bernardo Toledo, Joana Silva, Carina Trindade, Délio Raimundo, Marco F. L. Lemos

**Affiliations:** 1 MARE-Marine and Environmental Sciences Centre & ARNET—Aquatic Research Network Associated Laboratory, ESTM, Polytechnic of Leiria, 2520-641 Peniche, Portugal; 2 REQUIMTE/LAQV, Laboratório de Farmacognosia, Faculdade de Farmácia, Universidade do Porto, 4050-313 Porto, Portugal; 3 Departamento de Ecología Integrativa, Estación Biológica de Doñana (EBD), Consejo Superior de Investigaciones Científicas (CSIC), 41092 Sevilla, Spain; 4 Campotec IN, Silveira, 2560-393 Torres Vedras, Portugal

**Keywords:** biorefinery, fruit preservation, fungicides, marine biotechnology, seaweed antifungals, mycelial growth, spore germination

## Abstract

Fungal infections cause losses amounting to between 20 and 25% of the fruit industry’s total outcome, with an escalating impact on agriculture in the last decades. As seaweeds have long demonstrated relevant antimicrobial properties against a wide variety of microorganisms, extracts from *Asparagopsis armata*, *Codium* sp., *Fucus vesiculosus*, and *Sargassum muticum* were used to find sustainable, ecofriendly, and safe solutions against Rocha pear postharvest fungal infections. *Alternaria alternata*, *Botrytis cinerea*, *Fusarium oxysporum*, and *Penicillium expansum* mycelial growth and spore germination inhibition activities were tested in vitro with five different extracts of each seaweed (*n*-hexane, ethyl acetate, aqueous, ethanolic, and hydroethanolic). An in vivo assay was then performed using the aqueous extracts against *B. cinerea* and *F. oxysporum* in Rocha pear. The *n*-hexane, ethyl acetate, and ethanolic extracts from *A. armata* showed the best in vitro inhibitory activity against *B. cinerea, F. oxysporum*, and *P. expansum,* and promising in vivo results against *B. cinerea* using *S. muticum* aqueous extract were also found. The present work highlights the contribution of seaweeds to tackle agricultural problems, namely postharvest phytopathogenic fungal diseases, contributing to a greener and more sustainable bioeconomy from the sea to the farm.

## 1. Introduction

The development of agriculture is one of the most important tools to alleviate poverty and feed the fast-growing human population [1]. Fresh fruits, particularly pears, are a source of nutrients and active substances, whose role is very important for human health and wellbeing [2,3]. However, their production is often compromised by fungal infections, among other pathogens that are responsible for 20% of perennial losses, of which 10% are caused during postharvest stages [2,4,5]. Furthermore, these losses are tightly related to the global climate change, which is helping to increase the severity of plant diseases and the emergence of new pests [6,7].

Among the main fungi that affect pear production, *Alternaria alternata*, *Botrytis cinerea*, *Fusarium oxysporum*, and *Penicillium expansum* can be highlighted for causing worldwide losses in the total outcome of pear production [2,8,9]. 

Currently, to preserve crop yield and quality, the strategies used are almost entirely based on the use of synthetic pesticides to prevent, kill, or inhibit phytopathogens, providing a low-cost and temporary solution [10,11,12]. However, the excessive use of these compounds increases contaminations and risks for the environment [12,13] and the appearance of new resistant fungi or new endangered hosts, such as animals or even humans [10]. 

Considering the challenges that the agriculture industry must endure, while preserving the environment, the need to implement a sustainable agriculture is becoming an urgency. A sustainable agriculture promotes greener and safer practices, including the use of natural compounds to reduce the extensive use of synthetic fungicides [7]. In this context, several studies have been carried out to find alternatives, including biological control agents, formulations based on natural extracts, disinfecting agents, physical methods, among others [11,14]. However, despite the efforts made to combat postharvest fungal infections using an ecofriendly approach, none have been robust, effective, and cost-effective enough to replace the current solutions in pome fruit [9].

In the last decades, it has been increasingly reported that marine organisms synthesize a vast number of bioactive secondary metabolites with promising biotechnological applications [15]. It is estimated that seaweeds synthesize around 40,000 different compounds with antifungal activity, including polysaccharides, polyphenols, carotenoids, proteins, peptides, sterols, terpenes, and fatty acids [15,16,17]. Throughout the last century, both in vitro and in vivo assays were carried out (making use of mycelium, spores, and infected plants), showing variations in the antimicrobial activities depending on the seaweed (influenced by environmental and biological factors), the extraction method, and the solvents used [17]. It has been proven that different seaweed macerates and extracts are able to fight phytopathogenic fungi not only inhibiting or reducing the growth of their vegetative hyphae or attacking them by lytic enzymes but also inhibiting the germination of their conidia (mycostatic activity) [18]. In addition, they can induce the expression of certain genes in plants, promoting the activation of defense signaling pathways, leading to a better response of plants against different fungal diseases [18].

Portugal is a country with great diversity and richness; at least 246 species of Rhodophytes, 98 Phaeophytes, and 60 Chlorophytes have been described [19]. *Fucus vesiculosus* Linnaeus 1753 and *Codium* sp. Stackhouse, 1797 [20] are some examples of the seaweed species present in the Portuguese coast. Furthermore, overfishing, pollution, globalization, and climate change are promoting the emergence of invasive seaweed in the Portuguese coast, such as *Asparagopsis armata* (Harvey) and *Sargassum muticum* (Yendo) Fensholt [20,21,22]. These species have previously shown antimicrobial activities [17,18,23] and thus may possess great potential to act as a sustainable and ecofriendly source of antifungals. Moreover, in the case of invasive seaweeds, their harvest will also help to restore the affected ecosystems, while creating an added value to this biomass, contributing to a bio-based economy.

Thus, the main purpose of this work is to find more sustainable, ecofriendly, and safer solutions against postharvest fungal infections in Rocha pear long-term conservation, while adding value to the seaweeds from the Portuguese coast. 

## 2. Materials and Methods

### 2.1. Harvesting of Seaweeds and Extraction of Compounds

Four different seaweed species were used in this study and all collected in Portugal: *S. muticum,* hand-collected at the intertidal zone in Praia do Norte, Viana do Castelo (41.698517, −8.854803) in 2019; *F. vesiculosus,* hand-collected at the intertidal zone in Figueira da Foz (40.118368, −8.829818) in 2021; *A. armata*, hand-collected by SCUBA diving in Berlenga Natural Reserve, Peniche (39.410169, −9.514052) in 2021; and *Codium* sp., obtained from an aquaculture, Algaplus (Ílhavo, Portugal). Fresh biomasses were washed to remove encrusting materials, detritus, sand, and other contaminants and then dried in a wind tunnel at 25 °C for no longer than 36 h and milled to powder using a blade mill (particle size with an average less than 0.25 mm). Dried seaweeds were stored in sealed bags in the dark at room temperature, and controlled humidity until use.

Each seaweed was subjected to five different extractions using five solvents: *n*-hexane (VWR Chemicals BDH^®^, Rosny-sous-Bois-cedex, France), ethyl acetate (VWR Chemicals BDH^®^, Rosny-sous-Bois-cedex, France), ultrapure water, ethanol 99.5% (Aga, Portugal), and ethanol:water (ratio 75:25). Two liters of each solvent were incubated in agitation with 100 g of the seaweed at 625 rpm using an automatic shaker (Velp Scientifica, OHS 20 digital) for 4 h at room temperature and protected from light. The extracts were then vacuum-filtered using a qualitative filter paper, 415 (particle retention 12–15 µm, VWR), with the exception for the extraction with water, where a centrifugation (Centrifuge 5810 R, Eppendorf, Hamburg, Germany) at 10 °C for 15 min at 3220 × *g* was performed. For all of them, except the aqueous extraction, the solvent was evaporated by rotary evaporator (Heidolph Laborata 4000, Schwabach, Germany) (water bath at 40 °C). Finally, to ensure that those extracts were completely dried without any residual solvents, speed vac equipment (Eppendorf, Concentrator Plus) was used to evaporate any residual solvent present. In the case of the extraction with water, after centrifugation, the supernatant was collected, frozen at −80 °C, and lyophilized (CoolSafe Freeze Dryer, ScanVac, Frilabo, Barcelona, Spain). All the dried extracts were kept at 4 °C until further use. 

For the in vitro assays, the resuspension of the extracts at 100 mg/mL (stock solution) was performed using aseptic conditions, except for the aqueous extract of *A. armata*, which was resuspended at 50 mg/mL. For all the extractions, except for the aqueous ones, dimethylsulfoxide (DMSO, CARLO ERBA Reagents, Cornaredo, Italy) was used as resuspension solvent, while sterile ultrapure water was used for all the aqueous extracts. After resuspension, the extracts were stored at −20 °C until use. For the in vivo assays, the extracts were dissolved in distilled water (sterile) at 1 mg/mL at the time of the assays.

### 2.2. In Vitro Assays

#### 2.2.1. Mycelial Growth Inhibition Activity

To carry out the tests, the poisoned food technique was employed as described by Xu et al. [24], with minor modifications. The extracts (*n*-hexane, ethyl acetate, ultrapure water, ethanol, and ethanol:water) at 100 mg/mL were incorporated in Potato Dextrose Agar (PDA) (Merk KGaA, Darmstadt, Germany) to obtain a final concentration of 0.1, 0.5, and 1 mg/mL in Petri dishes of 55 mm. Mycelial plugs of 5 mm diameter were collected from the edge of fresh cultures of each fungal strain: *A. alternata*, *B. cinerea, F. oxysporum*, and *P. expansum (*Westerdijk Fungal Biodiversity Institute, Netherlands) and placed on the center of the Petri dishes. Three controls were performed for each assay: a growth control (fungi inoculated in PDA medium), a vehicle control (fungi inoculated in PDA medium supplemented with DMSO at 0.1, 0.5, and 1 mg/mL), and a growth inhibition control (fungi inoculated in PDA medium supplemented with amphotericin B at 30 µg/mL (Laborspirit, Lda., Lisbon, Portugal)). The cultures were incubated at 24 °C in the presence of light for *B. cinerea*, *A. alternata*, and *P. expansum* and 21 °C in the absence of light for *F. oxysporum* until the limit of the plate was reached by the mycelial growth in all the replicates of one of the conditions tested (including the controls). For each condition, three replicates were made, and daily radial growth measurements (mm) were carried out.

The inhibition of the mycelial growth was calculated as follows: (growth control (mm)–treatment (mm))/(growth control (mm)−vehicle control (mm)).

#### 2.2.2. Spore Germination Inhibition Activity

For the microdilution method, fungi were incubated in PDA (*A. alternata*, *F. oxysporum*, and *P. expansum*) or PDA using 1/10 of the normal concentration (*B. cinerea*) at 24 °C in the presence of light for *A. alternata*, *B. cinerea*, and *P. expansum* and 21 °C in the absence of light for *F. oxysporum*. In addition, *A. alternata*, *B. cinerea*, and *F. oxysporum* were incubated with sterilized pear tree branches (around 2 cm) placed on the agar surface. The spores were collected using sterile ultrapure water with Tween 20 (VWR International, Leuven, Belgium) at 0.1%. The surfaces of the Petri dishes were washed and, in the case of *A. alternata*, *B. cinerea*, and *F. oxysporum*, were also scraped with an inoculation loop. For these last three fungi, the solution collected was filtered with several layers of sterile gauze, so the maximum quantity of mycelium was eliminated from the sample. Afterward, the concentration of the solution was adjusted to a final concentration of 1.5 × 10^5^ spores/mL in the 96-well microplate, as indicated by EUCAST protocol (E.DEF 9.3.2) [25], using the medium Roswell Park Memorial Institute—1640 (RPMI-1640, Sigma-Aldrich, St. Louis, MO, USA) to complete the final volume (200 µL).

Three different concentrations of the extracts (*n*-hexane, ethyl acetate, ultrapure water, ethanol, and ethanol:water) were tested (0.1, 0.5, and 1 mg/mL). A positive control of amphotericin B (Laborspirit, Lda., Lisbon, Portugal) was used at 2 µg/mL as well as a vehicle control (DMSO at 0.1, 0.5, and 1 mg/mL). The incubation was carried out during 24 h (*F. oxysporum* and *P. expansum*), 48 h (*A. alternata*), and 96 h (*B. cinerea*) at 21 °C in the darkness (*F. oxysporum*) or 24 °C in the presence of light (*A. alternata, B. cinerea*, and *P. expansum*). 

The Minimum Inhibitory Concentration (MIC) was determined as the lowest extract concentration with no visible growth detected using an inverted trinocular fluorescence microscope (ZEISS Vert.A1, Carl Zeiss, Göttingen, Germany) [25]. The Minimum Fungicidal Concentration (MFC) was determined by subculturing 80 µL of those wells with MIC into 6-well microplates (VWR Chemicals BDH^®^) with PDA (Merk KGaA, Darmstadt, Germany). The 6-well microplates were incubated for the same period and conditions as the 96-well microplates and read again using a Trinocular Stereo Microscope (ZEISS Stemi 2000-C, Carl Zeiss, Göttingen, Germany). The MFC was considered as the minimum concentration at which no visible growth was detected in the subcultured 6-well microplates [26,27]. The experiment was carried out in triplicate, and three independent replicates were performed.

### 2.3. In Vivo Assays

#### 2.3.1. Aqueous Extract Toxicity in Rocha Pear

The toxicity of the aqueous extracts at 1 mg/mL of *A. armata*, *Codium* sp., *F. vesiculosus*, and *S. muticum* was evaluated in Rocha pear. Rocha pears were cultivated and commercially harvested by CAMPOTEC IN (Silveira, Torres Vedras, Portugal). In vivo assays were performed based on the work of Nikkhah et al. (2017).

Pear fruits were washed with tap water, disinfected by immersing them in 2% of sodium hypochlorite for 2 min, rinsed with sterile water, and dried at room temperature. Two wounds per pear were made (5 mm in diameter and 3 mm in depth) in the equatorial region of the fruits. Pears were treated with the aqueous extracts, using 200 mL of extract solution per 100 g of fruit. For that, fruits were immersed into each extract solution (treatment) for 10 min and then air-dried at room temperature for two hours. A control was also made, consisting of the fruit wounded without any extract treatment. Then, the pears were incubated for 11 days at 22 °C in the absence of light and at 95% relative humidity (RH). The toxicity of the extracts in the pears was assessed by visual alterations in periods of 24 h for 11 days. Six replicates of each treatment were made, considering one replicate as one pear, and the assay was executed in a randomized design.

#### 2.3.2. Aqueous Extract Antifungal Activity 

A protective assay, using the aqueous extracts, was carried out. The cleaning, disinfection, wound performing, and treatment were performed as described in Section 2.3.1. Additionally, two controls were evaluated: the fruit wounded without any treatment and the wounded fruit immersed in water for 10 min and infected with the fungi. 

The inoculation was performed by adding 20 µL of a spore suspension of *F. oxysporum* and 40 µL of *B. cinerea*, both at 5 × 10^6^ spores/mL into each wound. The inoculated pears were dried at room temperature for 30 min and then incubated for 11 days (*B. cinerea*) and 7 days (*F. oxysporum*) at 22 °C in the dark and at 95% RH. The fungal culture and the spore collection were made as described in the spore germination inhibition assay (Section 2.2.2), but the collection of the spores was performed using only sterile ultrapure water. 

The fruit decay was assessed daily by measuring the radius of the decay halo in millimeters in every wound. Six replicates of each treatment were performed, considering one replicate as one pear, and the assay was executed in a randomized design.

### 2.4. FTIR-ATR Spectroscopic Analysis

Functional groups of the more relevant seaweed extracts (*n*-hexane, ethyl acetate, and ethanolic extract from *A. armata* and aqueous extract from *S. muticum*) were evaluated by the Fourier transform infrared spectroscopy attenuated total reflection (FTIR-ATR) technique. The FTIR analysis were carried out using an FT-IR UATR Two spectrometer (Perkin Elmer, MA, USA) in a range of 450–4000 cm^−1^ at a 4 cm^−1^ resolution with 64 scans. Each sample was analyzed two times.

### 2.5. Statistical Analysis

For the mycelial growth inhibition assays, linear regressions were calculated for the growth inhibition in mm of each condition, and its slope was considered as the growth inhibition rate (GIR) and then compared with the growth control rate of each assay (slope of the linear regression of the growth controls) (GraphPad Prism 8). For the in vivo assays, a generalized linear model (GLM) with a quasi-probability density function was performed with the equation y = log(x), using the variance as mean (R, version 4.1.3), where significant differences (* *p* < 0.05, ** *p* < 0.01, and *** *p* < 0.001) were considered [28]. The estimates of the controls, the time, and the treatments were added together (Value) and then transformed to remove the logarithmic (Linear Value), being able to obtain the percentage of the real effect of each treatment in comparison with the control (Real effect).

## 3. Results

### 3.1. Seaweed Extract Yields

The yields of the extractions (*n*-hexane, ethyl acetate, water, ethanol, and ethanol:water) of the four seaweeds are indicated in Table 1. The yield of the aqueous extracts should be highlighted as they represent the highest values for all seaweeds when compared with the remaining extracts. On the contrary, the extractions using *n*-hexane presented the lowest yields for all seaweeds, with only *F. vesiculosus* exceeding 1%. 

Furthermore, taking seaweeds into account, *Codium* sp. and *F. vesiculosus* have the best performance depending on the solvent, *A. armata* being the one with lower yields in all the extractions except for the ethanolic extract in which *S. muticum* had the lowest yield.

### 3.2. Mycelial Growth Inhibition Activity

Regarding the results obtained for the *A. armata* extracts against *B. cinerea*, as shown in Table 2, the ethyl acetate and hydroethanolic extracts at 1 mg/mL presented the highest inhibitory activity against this fungus, reaching inhibitions of 69.51% and 67.63%, respectively. Furthermore, the *n*-hexane extract at 1 mg/mL and ethanolic at 0.5 and 1 mg/mL, also shown relevant inhibitions accounting for 55.30%, 51.26%, and 50.85%, respectively. *F. vesiculosus* also demonstrated inhibitory activities with the *n*-hexane extract at 1 mg/mL (42.59%) and the aqueous extract at 0.1 and 0.5 mg/mL (60.15 and 43.29%, respectively).

Additionally, the ethyl acetate extract at 1 mg/mL from *A. armata* also presented inhibitory activity against *P. expansum*, inhibiting its mycelial growth by 44.59%.

However, none of the extracts showed relevant inhibitory activity against the mycelial growth of *F. oxysporum* or *A. alternata.* It is worth mentioning that in the case of *A. alternata*, an increase in the mycelial growth was observed in almost every extract from all the seaweeds. All the results obtained are displayed in Table S1.

### 3.3. Spore Germination Inhibition Activity

Concerning the spore germination, none of the extracts from the four seaweeds exhibited inhibitory effects against *A. alternata* or *P. expansum*, because after 48 h or 24 h (necessary time following the growth control to completely germinate the spores, respectively), all the microplate test wells were full of mycelia. 

**Figure 1:**
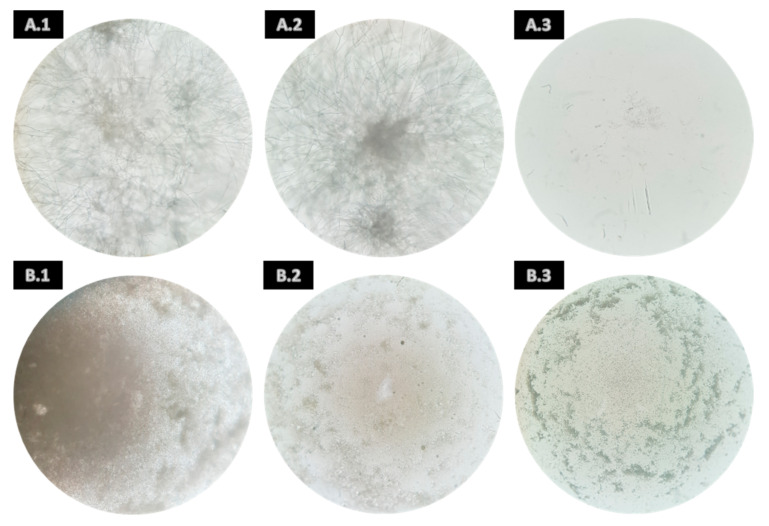
Representative examples of the spore germination minimum inhibitory concentration (MIC) of *Botrytis cinerea* by the *n*-hexane, ethyl acetate, and ethanolic extracts of *Asparagopsis armata*. Where (**A.1**): DMSO control (0.5 mg/mL); (**A.2**): DMSO control (1 mg/mL); (**A.3**): amphotericin B control (2 µg/mL); (**B.1**): *n*-hexane (1 mg/mL); (**B.2**): ethyl acetate (0.5 mg/mL); and (**B.3**): ethanol (0.5 mg/mL).

Concerning *B. cinerea*, some extracts of *A. armata* showed inhibitory activity against its spore germination. As can be seen in Table 3, the *n*-hexane extract presented MIC at 1 mg/mL, and the ethyl acetate and ethanolic extracts presented MIC at 0.5 mg/mL (Figure 1(B.1–B.3), respectively); in addition, inhibitory activity was verified at 1 mg/mL (MIC × 2) in both extracts (Figure S1). Furthermore, MFC was observed in the *n*-hexane and ethyl acetate extract samples at 1 mg/mL (Figure S1), and in ethanolic extract samples, inhibitory activity was seen at 0.5 mg/mL (MFC) and 1 mg/mL (MFC × 2) (Figure S1). The figures show the differences between the DMSO controls and the inhibitory extracts at the respective concentrations, in which no visible mycelium growth was found in the extract samples in comparison with a fully covered well or plate in the case of the controls. 

**Figure 2:**
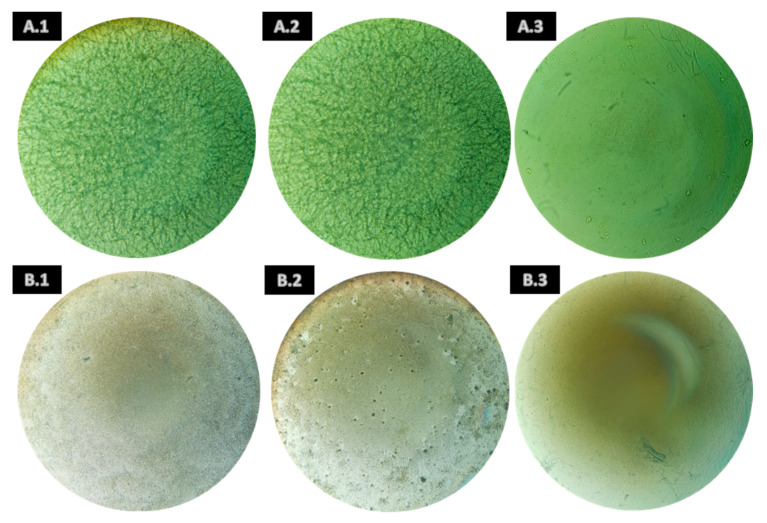
Representative examples of the spore germination minimum inhibitory concentration (MIC) of *Fusarium oxysporum* by the *n*-hexane, ethyl acetate, and ethanolic extracts of *Asparagopsis armata*. Where (**A.1**): DMSO control (0.5 mg/mL); (**A.2**): DMSO control (1 mg/mL); (**A.3**): amphotericin B control (2 µg/mL); (**B.1**): *n*-hexane (0.5 mg/mL); (**B.2**): ethyl acetate (0.5 mg/mL); and (**B.3**): ethanol (1 mg/mL).

Likewise, for *F. oxysporum*, only extracts from *A. armata* presented MIC (Table 3) and *n*-hexane and ethyl acetate extracts at 0.5 mg/mL (Figure 2(B.1) and (B.2), respectively), also showed activity at 1 mg/mL (MIC × 2) (Figure S2). Furthermore, the ethanolic extract presented MIC at 1 mg/mL (Figure 2(B.3)). Regarding the MFC, it was observed for *n*-hexane and ethyl acetate at 1 mg/mL (Figure S2). However, after 7 days at the end of the assay, they were fully covered with mycelia, showing that only a slowdown of the germination of spores was reached.

### 3.4. In Vivo Assays

#### 3.4.1. Aqueous Extract Toxicity in Rocha Pear

**Figure 3:**
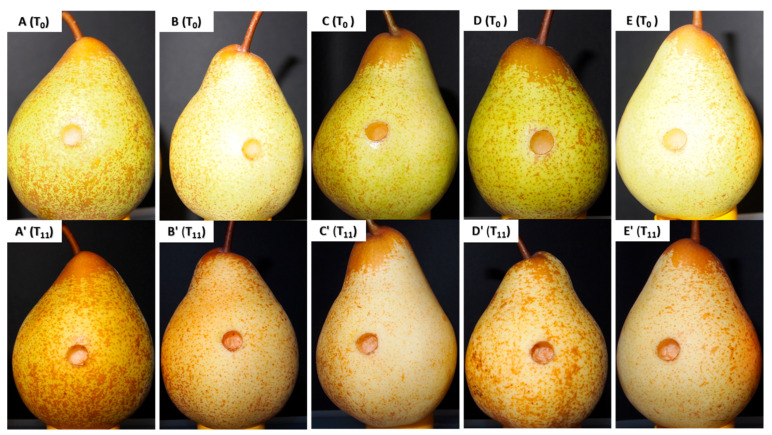
Illustrative examples of the pears immersed in the aqueous extracts of the four seaweeds at 1 mg/mL at the beginning of the experiment (T_0_) and after 264 h of incubation (T_11_). (**A**,**A’**): *Asparagopsis armata* aqueous extract; (**B**,**B’**): *Codium* sp.; (**C**,**C’**): *Fucus vesiculosus*; (**D**,**D’**): *Sargassum muticum;* and (**E**,**E’**): wounded pear without treatment (control).

The aqueous extracts from *A. armata*, *Codium* sp., *F. vesiculosus*, and *S. muticum* at 1 mg/mL did not show any detectable visual changes in any of the tested pears after 264 h (11 days) of exposure to the extract, as illustrated in Figure 3. Only the increment of ripening in the fruit was observed in all the samples, including the control, which was noticed by the softness of the tissues and the color alteration of the pears from green to brownish yellow (Figure 3).

#### 3.4.2. Aqueous Extract Antifungal Activity

**Figure 4:**
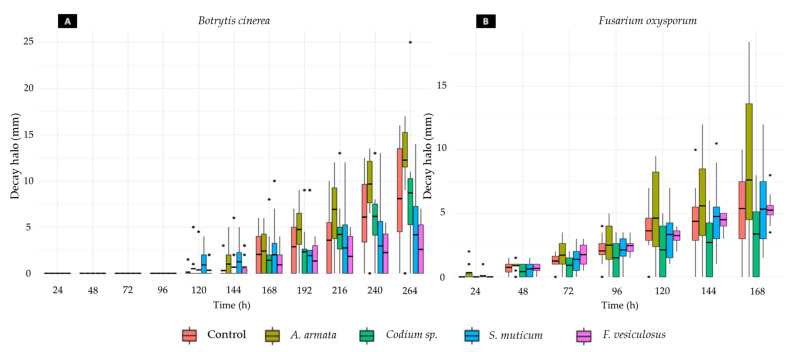
Boxplot displaying the distribution of the data obtained after immersing Rocha pear in the aqueous extracts of *Asparagopsis armata*, *Codium* sp., *Fucus vesiculosus,* and *Sargassum muticum* at 1 mg/mL and inoculating the fruits with 5 × 10^6^ spores/mL of *Botrytis cinerea* (**A**) or *Fusarium oxysporum* (**B**). The growth control is also represented. Outliers are indicated with •.

Concerning the data collected during both assays (Rocha pear infected with *B. cinerea* and *F. oxysporum*), a great variance among the data obtained for each condition was found (Figure 4). Considerable differences between the growth of the halos in the wounds of different pears and between different wounds of the same pear were noticed.

Regarding the differences obtained in the disease halo measurements of the different replicates of each condition, as can be seen in Table 4, these differences are exacerbated due to inoculated wounds that did not present any decay halo. This tendency was observed in most of the conditions, except for the pears immersed in *F. vesiculosus* and *S. muticum* and inoculated with *F. oxysporum.* However, in the case of *B. cinerea,* the pears treated with the same aqueous extracts presented seven pears without a decay halo in the *F. vesiculosus* treatment and six pears in the *S. muticum* treatment.

**Figure 5:**
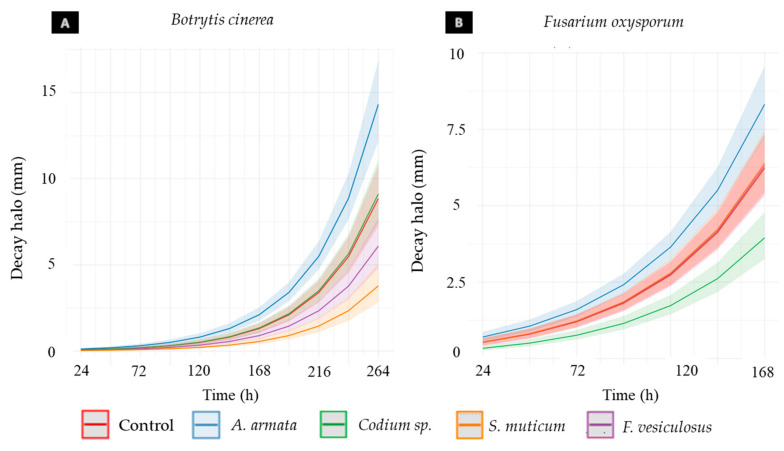
Logarithmic curve of the decay halo growth in millimeters (mm) over the test time (h), after treating Rocha pears with aqueous extracts of *Asparagopsis armata*, *Codium sp.*, *Fucus vesiculosus*, and *Sargassum muticum* at 1 mg/mL and inoculating the fruits with a spore suspension of *Botrytis cinerea* (**A**) or *Fusarium oxysporum* (**B**). The growth control is also represented for each fungus. The confidence interval (CI95) of the data is illustrated by the translucent areas around the lines of the same colors.

As illustrated in Figure 5A, the Phaeophyta seaweeds show significant effects on the pears infected by *B. cinerea*, slowing down the decay halo growth by 34.15% with *F. vesiculosus* treatment and 57.21% with *S. muticum* treatment (Table 5). All the statistics values are detailed in Table S2. Furthermore, a significant stimulation of the development of the fungal infection was observed caused by the treatment with *A. armata.* Regarding the *F. oxysporum* infection (Figure 5B), the only seaweed extract that showed a significant inhibition was *Codium* sp.*,* which slowed down the decay halo growth by 36.60% (Tables Table 5 and Table S3). However, a significant growth stimulation of the decay halo was again observed in the pears treated with *A. armata* aqueous extract.

### 3.5. Fourier Transform Infrared Spectroscopy (FTIR-ATR)—Analysis

FTIR-ATR was used to identify the main functional groups of the seaweed extracts with the most promising results (*n*-hexane, ethyl acetate, and ethanolic extract from *A. armata* and the aqueous extract from *S. muticum*). The main peaks identified in the extract spectra are displayed in Table 6, and the original spectra can be observed in Figure S3.

## 4. Discussion

The emergence of new fungal phytopathogenic species all over the world and the increase in fungal resistance to the current solutions, such as synthetic pesticides, is one of the main challenges that the agriculture industry is currently facing [1,6,7,10,39]. This affects the yield of fruit production, causing great losses during the postharvest stages [2,4]. In this context and accounting for the negative impact that synthetic fungicides have not only on the environment but also on human and animal health [10,12,13], the search for novel, safer, and more sustainable solutions is of extreme importance.

Due to the characteristics of the marine environment, seaweeds are in constant contact with several microorganisms, having often developed strong secondary metabolite chemical defenses, allowing them to survive in the presence of several pathogens [40]. Seaweeds (Chlorophyta, Phaeophyta, and Rhodophyta) have shown antifungal activities against several species as reviewed in Vicente et al. (2021).

### 4.1. In Vitro Assays

In the present work, a screening in vitro was performed to assess the potential of five different extracts from *A. armata* and *S. muticum* (two invasive species on the Portuguese coast) and *Codium* sp., and *F. vesiculosu*s against four fungi (*A. alternata*, *B. cinerea*, *F. oxysporum*, and *P. expansum*) responsible for significantly decreasing the total pear production outcome [8,9,41,42,43,44]. The extracts were tested against the mycelial growth and spore germination of the previously mentioned fungi, because the mycelium is the vegetative part of the fungi, while the spores can act as resistant forms, allowing them to survive for long periods in stressful conditions, and they are likely to have different responses to the same compounds or concentrations [45,46].

Concerning the mycelial growth inhibition (Table 2), the results obtained by the organic extracts of *A. armata* (*n*-hexane, ethyl acetate, ethanolic, and hydroethanolic extracts) should be highlighted, reaching inhibitions higher than 65% against *B. cinerea* and 40% against *P. expansum* in the case of the ethyl acetate extract. It has been reported that these activities can be related to several compounds, including fatty acids or terpenes (*n*-hexane and ethyl acetate extracts) [17,19,47] and phenolic compounds (ethyl acetate, ethanolic, and hydroethanolic extracts) [17,48]. In addition, the inhibitory activity higher than 60% by the aqueous extract of *F. vesiculosus* against *B. cinerea* is likely related to polysaccharides commonly present in brown seaweeds, such as laminarin fucoidans or alginates and phlorotannins, both compounds having previously shown activity against phytopathogenic fungi [49,50]. For the *n*-hexane extract, inhibitory activity against *B. cinerea* (higher than 40%) was also found and is likely to be related to lipophilic compounds [51]. It is worth mentioning that the aqueous extract of *F. vesiculosus* presented decreasing inhibitory activities with an increasing extract concentration. This decrease in activity may have been caused by the decrease in bioavailability of the bioactive compounds in the medium with the increase in the concentration, as Benoit et al. (2001) described in the case of Hg bioavailability for the bacteria *Desulfobulbus propionicus* with the increase in sulfide concentration in the medium [52]. Another possibility may be related to the complex composition of the extracts, where antagonistic effects between the compounds of the aqueous extracts may be present at the highest concentration. Meletiadis et al. (2007) studied the synergistic and antagonistic interactions of the combination of three different antifungals (amphotericin B, voriconazole, and caspofungin) and demonstrated that at higher concentrations of amphotericin B, the antagonistic interactions increased, leading to a decrease in drug effectivity [53]. 

Moreover, it was expected that the commercial fungicide Amphotericin B would show a higher inhibition than the seaweed extracts (Table 2) because the components that have antifungal activity in the extracts are not purified; they are complex mixtures with all the metabolites that are soluble in the solvent. Nevertheless, it is worth mentioning that the activity of the ethyl acetate and hydroethanolic extracts of *A. armata* against *B. cinerea* reached higher inhibitions than their Amphotericin B control, which further highlights the potential of these seaweeds. 

Regarding spore germination inhibition, *A. armata* was the only seaweed with extracts with relevant inhibitory results (Table 3). Inhibitory activity was found against *B. cinerea* and *F. oxysporum*, presenting MIC by the *n*-hexane, ethyl acetate, and ethanolic extracts of *A. armata* and MFC by the *n*-hexane and ethyl acetate extracts. Nevertheless, the plates representing *F. oxysporum,* after one week of incubation, were fully covered by mycelia, indicating that only a slowdown in the spore germination was achieved using these extracts. In addition, the ethanolic extract presented MFC against *B. cinerea*. The obtention of MIC but not MFC in some concentrations may be related to the ability of the extracts to possibly slow down the germination rate of the spores, not affecting their viability. Thus, when the fungal spores are cultured again in a PDA medium without an extract, the spores find suitable conditions to germinate. Another hypothesis is related to the capacity of several species of fungi, such as *F. oxysporum*, to form different types of spores with different resistances in the same colony, with the possibility that the inhibitory extracts affect only one type of spore and are unable to affect the macroconidia (more resistant than microconidia) [54]. Moreover, it could be observed that although *F. oxysporum* and *B. cinerea* spore germination seemed to be susceptible to mostly the same extracts, a completely different response was seen in *A. alternata* and *P. expansum*, because none of the extracts affected the spore germination, which may be related to the great spore diversity among fungal species, influencing their resistance to stress [46]. 

Thus, the most promising in vitro results are those observed in *A. armata* extracts, within which the *n*-hexane, ethyl acetate, and ethanolic ones showed inhibition for both mycelial growth and spore germination against *B. cinerea*, against the spore germination of *F. oxysporum*, and against the mycelial growth of *P. expansum* (ethyl acetate extract). Given the results obtained for the *n*-hexane and ethyl acetate extracts in the FTIR-ATR analysis (Table 6), the inhibitory activity of these extracts might be associated with the presence of volatile halogenated compounds, specifically brominated or chlorinated compounds, as suggested by the presence of the peaks at 720 cm^−1^ in both spectra [35]. *A. armata* is known to synthetize these kinds of compounds, having already been described as having antifungal activities against phytopathogenic fungi, among other bioactivities [23,55]. Furthermore, the peak at 1650 cm^−1^ observed in both extracts suggests the presence of lipophilic amides, which also might be responsible for the antifungal activity detected, once they have already been hypothesized as being responsible for antifungal activities of natural extracts [56,57]. In addition, the peaks at 1710 cm^−1^ (*n*-hexane) and 1709 cm^−1^ (ethyl acetate) raise the possibility of the existence of fatty acids in the extracts [32], some of which might have antifungal activities [17,22]. In addition to the possibility of also containing antifungal fatty acids (1174 cm^−1^, 1373 cm^−1^, and 2924 cm^−1^), as well as the same amide-associated signal at 1649 cm^−1^, there is also the possibility of occurrence of ethanol-soluble, low-molecular-weight carbohydrate derivatives (1095 cm^−1^ and 1035 cm^−1^) [38], which might also be responsible for the antifungal activities detected [58,59]. 

Accordingly, although there is a lack of information regarding the antifungal activity against filamentous fungi of this seaweed, the presence of compounds able to inhibit bacteria, yeasts, virus, and protozoa growth has been demonstrated in several works, as reviewed in Félix et al. (2021). Due to the extensive number of studies with Phaeophyta reporting activity against phytopathogenic fungi [17], more inhibitory activities from *F. vesiculosus* and *S. muticum* would be expected in comparison with *A. armata*, once *S. muticum* did not show any inhibitory activity against mycelial growth or spore germination and *F. vesiculosus* showed no activity against spore germination. Nevertheless, it is precisely the difference in the number of studies using Phaeophyta and Rhodophyta species that might be the origin of the lack of antifungal activity reported for *A. armata*.

### 4.2. In Vivo Assays

Following the main objective of this work, the search for greener and safer solutions against postharvest phytopathogenic fungi in Rocha pear, preliminary in vivo assays, was performed in Rocha pear to assess the growth inhibitory activity of the aqueous extracts of *A. armata*, *Codium* sp., *F. vesiculosus*, and *S. muticum*. Given that no inhibitory results were obtained against *A. alternata* or *P. expansum* in the in vitro screening against the spore germination, only the other two fungal pathogens (*B. cinerea* and *F. oxysporum*) were considered for the preliminary in vivo assays. 

Despite the results obtained in the in vitro assays, where only the *F. vesiculosus* aqueous extracts presented inhibitory activity, the aqueous extracts were selected for this task due to their compatibility with the food industry [60] and higher extraction yields obtained for these extracts when compared to the other solvents used, enabling its potential industrial use. To evaluate the potential toxicity of the extract on fruits, a higher test concentration was chosen, 1 mg/mL. The results obtained show no visual signs of toxicity (Figure 3), allowing the utilization of this concentration for the assay for all treatments. 

Regarding the inhibitory activity assay of the aqueous extracts in Rocha pear against the selected fungal species, the variability observed in the results obtained (size of necrotic halos) was mostly verified to be caused by the presence of pears without halos (P0s) in almost every treatment and control for both fungi (Figure 4). Once P0s were also observed in the fungal growth controls, other explanations rather than extract effect only should be considered, including different fruit ripening stages, despite the same batch and appearance being used. In fact, it was observed that the ripening stage determined the rapidity at which the decay halo grew and the severity the fungal infection reached, with those with more advance ripening stages being the more susceptible samples to the infection. According to these observations, Petrasch et al. (2019) confirmed that the fruit ripening stage has a high impact on the success of the infection of *B. cinerea* and *Fusarium acuminatum* [61]. The presence of several P0s along treatments demonstrates the great variability that exists among fruits collected from the same orchard at the same time, highlighting the importance of using a higher number of replicates of each condition, as suggested in other studies [62,63,64], in order to prove the results obtained in this study. Nevertheless, given a randomized distribution of pears, the higher number of P0s in *F. vesiculosus* and *S. muticum* (Table 4), may hint toward a potentially more effective effect of these extracts reducing the infection.

Contrary to the results obtained in the in vitro assays, *A. armata* showed growth stimulant activity for both fungi, while *Codium* sp. presented a statistically significant inhibition of 36.60% against *F. oxysporum* (Table 5). However, looking for a potential application in the agriculture industry, the aqueous extract presenting the most interesting inhibition was *S. muticum* against *B. cinerea*, reaching an inhibition higher than 50% when compared to the control (Table 5). Nevertheless, a cautionary approach to these values should be taken as they were calculated considering the P0s obtained, which may or may not be experimental design shortcomings, as discussed above. It should be noted that although it is not reflected in the inhibition percentage, those pears that presented decay halos after the application of the *S. muticum* aqueous extract showed smaller halos than those in the control, while in the case of *Codium* sp., the inhibitory activity that was observed was exclusively dependent on the P0 presence, because no differences were noticed among the maximum radii of the decay halos that grew in the samples treated and the ones of the control (Table 4). In addition, to account for the influence of the extract concentration in the in vitro tests, other concentrations should be tested in fruit assays to fully understand the behavior of the extracts and find the most promising extracts/concentrations. This potential activity of the *S. muticum* aqueous extract may be related to the presence of sulfated polysaccharides, which have been widely described in the bibliography as soluble compounds with antifungal activities [17], because three peaks were identified in the FTIR-ATR spectrum that could indicate their presence, particularly 1411 cm^−1^ (sulfated groups, S=O stretching), 1027 cm^−1^ (stretching vibrations of the glycosidic C–O bond), and the peaks between 1228 cm^−1^ (sulfated esters, asymmetric stretching S=O) (Table 6) [17,30]. 

Even though the main goal of this work is to find antifungal activity in the seaweed extracts selected against *A. alternata, B. cinerea*, *F. oxysporum*, and *P. expansum*, their application in fruits foresees several variables that can interfere and influence the results. It is possible that the inhibitory results found in this study are not directly related to fungal inhibition, but instead because they can act directly in the fruits, activating defense pathways of the pears, known as priming events [65]. In this context, some compounds that are likely to be present in the aqueous extracts due to their polarity, such as polysaccharides, have been proven to promote the activity of certain defense enzymes in plants, including, peroxidases (POD), polyphenol oxidases (PPO), and phenylalanine ammonia lyases (PAL) [50]. This is also in line with what has been shown by Sbaihat et al. (2015), who reported that an elicitor extracted from *Sargassum fusifurme* proved no antifungal activity in an in vitro assay against *B. cinerea*, but, on the contrary, in in vivo assays it was demonstrated to have a significant protection in tomato plants (*Solanum lycopersicum*) against this phytopathogenic fungi [66]. Therefore, it is important to analyze the behavior of key enzymes in the in vivo assays for a better understanding of what is occurring after the treatment with the extracts and the fungal inoculation. This will allow a better understanding of the associated mechanism of action and thus to find the optimal formula to apply in these fruits to prevent diseases in a postharvest stage.

## 5. Conclusions

The present work allowed to reveal the promising antifungal activities of several extracts (*n*-hexane, ethyl acetate, and ethanolic) from A*. armata* extracts in in vitro assays against the mycelial growth and spore germination of *B. cinerea, F. oxysporum,* and *P. expansum*. The *S. muticum* aqueous extract was also found to have great potential to inhibit the infection caused by *B. cinerea* in Rocha pear. Furthermore, in both in vitro and in vivo assays, stimulant mycelial growth activities were found in extracts from the four seaweeds tested, indicating that the biotechnological potential of these extracts may not be solely limited to antifungal bioactivity but can be further explored aiming at different purposes.

This study highlights several extracts from these algal biomasses that should be further studied and can be used as potential sustainable and ecofriendly strategies to combat postharvest fungal diseases that affect the conservation of Rocha pears.

## Figures and Tables

**Table 1:** Percentages of extraction yields of *Asparagopsis armata*, *Codium* sp., *Fucus vesiculosus*, and *Sargassum muticum* for the five different solvents used (*n*-hexane, ethyl acetate, water, ethanol, and ethanol:water).

Extraction Yields (%)				
Solvents	*A. armata*	*Codium* sp.	*F. vesiculosus*	*S. muticum*
*n-*hexane	0.08	0.62	4.14	0.37
Ethyl acetate	0.29	1.19	4.62	1.25
EtOH:H_2_O	2.00	20.15	20.64	6.66
EtOH	2.01	5.96	8.60	1.22
H_2_O	7.91	49.78	28.69	8.99

**Table 2:** Relevant growth inhibition rates (GIRs) in mm/h obtained in the poisoned food technique assay for the *n*-hexane, ethyl acetate, ethanolic, and hydroethanolic extracts of *Asparagopsis armata* at 0.5 and 1 mg/mL and the *n*-hexane and aqueous extracts of *Fucus vesiculosus* against *Botrytis cinerea* and *Penicillium expansum*. The GIR is followed by the confidence interval [CI95] and the inhibition percentage in comparison with the control growth rate (CGR), which is also indicated for each assay.

Relevant Growth Inhibition Rates				
*B. cinerea–A. armata*				
GIR (mm/h)	Extracts	Concentration(mg/mL)	GIR (mm/h) (CI95)	Inhibition (%)
	*n-*hexane	1	0.1206 (0.1014, 0.1398)	55.30
	Ethyl acetate	1	0.1516 (0.1425, 0.1607)	69.51
	EtOH	0.5	0.1118 (0.0987, 0.1249)	51.26
		1	0.1109 (0.0898, 0.1319)	50.85
	EtOH:H_2_O	1	0.1475 (0.1400, 0.1549)	67.63
	Amphotericin B	30 µg/mL	0.1417 (0.1345, 0.1488)	64.97
GCR (mm/h)	0.2181 (0.2040, 0.2321)			
*B. cinerea–F. vesiculosus*				
	Extracts	Concentration (mg/mL)	GIR (mm/h) (CI95) (inhibition %)	Inhibition (%)
GIR (mm/h)	*n*-hexane	1	0.0977 (0.0859, 0.1095)	42.59
	H_2_O	0.1	0.1380 (0.1167, 0.1592)	60.15
		0.5	0.0993 (0.0728, 0.1258)	43.29
	Amphotericin B	30 µg/mL	0.1711 (0.1587, 0.1834)	74.56
GCR (mm/h)	0.2294 (0.2201, 0.2387)			
*P. expansum–A. armata*				
	Extracts	Concentration (mg/mL)	GIR (mm/h) (CI95) (inhibition %)	Inhibition (%)
GIR (mm/h)	Ethyl acetate	1	0.0272 (0.0235, 0.0309)	44.59
	Amphotericin B	30 µg/mL	0.0447 (0.0437, 0.0455)	74.07
GCR (mm/h)	0.0610 (0.0602, 0.0618)			

**Table 3:** Minimum inhibitory concentration (MIC) and minimum fungicidal concentration (MFC) shown by the *n*-hexane, ethyl acetate and ethanolic extracts from *Asparagopsis armata* against the spore germination of *Fusarium oxysporum* and *Botrytis cinerea*.

*A. armata* Extracts with Spore Germination Inhibition Activity				
	*B. cinerea*		*F. oxysporum*	
Solvents	MIC (mg/mL)	MFC (mg/mL)	MIC (mg/mL)	MFC (mg/mL)
*n-*hexane	1	1	0.5	1
Ethyl acetate	0.5	1	0.5	1
EtOH	0.5	0.5	1	-

**Table 4:** Minimum and maximum radii of the halo decay, measured in millimeters (mm), at the end of the incubation of 11 days (264 h) for *Botrytis cinerea* and 7 days (168 h) for *Fusarium oxysporum*), after immersion of the Rocha pears in the aqueous extracts of *Asparagopsis armata*, *Codium* sp., *Fucus vesiculosus*, and *Sargassum muticum* at 1 mg/mL and inoculation of the fruits with *Botrytis cinerea* or *Fusarium oxysporum*. The quantity of pears that did not show any decay halo (P0) is also indicated. The data of the growth control are also indicated.

		*B. cinerea*			*F. oxysporum*			
	Minimum (mm)	Maximum (mm)	Mean ± SD	Number of P0s	Minimum (mm)	Maximum (mm)	Number of P0s	Mean ± SD
Control	0	16	8.083 ± 5.780	3	0	10	2	5.375 ± 3.196
*A. armata*	0	17	12.25 ± 4.361	1	0	18.5	1	7.625 ± 5.643
*Codium* sp.	0	25	8.708 ± 5.974	1	0	8	4	3.375 ± 2.754
*F. vesiculosus*	0	14	4.167 ± 5.417	7	2	12	0	5.333 ± 3.164
*S. muticum*	0	7	2.583 ± 2.753	6	3.5	8	0	5.250 ± 1.127

**Table 5:** Estimate model table for the relationship among decay halo growth, time, and treatment with the seaweed aqueous extracts (*Asparagopsis armata*, *Codium sp.*, *Fucus vesiculosus*, and *Sargassum muticum*) against *Botrytis cinerea* and *Fusarium oxysporum*. The ratio at which the curves vary depending on the treatments used and the influence of time (Estimate) are represented, followed by the standard error (Std. Error) and the percentage of the stimulant (positive) or inhibitory (negative) effect on the fruit decay in comparison with the control. Furthermore, significant differences are indicated with asterisks, where * *p* < 0.05 and *** *p* < 0.001.

	*B. cinerea*			*F. oxysporum*		
	Estimate	Std. Error	Real Effect (%)	Estimate	Std. Error	Real Effect (%)
Control	−3.077	0.236	0.000	−1.055	0.131	0.000
*A. armata*	0.481	0.121	61.799 ***	0.289	0.098	33.497 *
*Codium* sp.	0.030	0.134	3.012	−0.456	0.118	−36.603 *
*F. vesiculosus*	−0.373	0.149	−31.145 *	0.012	0.104	1.196
*S. muticum*	−0.849	0.174	−57.213 ***	0.025	0.104	2.502
Observations	658			418		
R^2^ Nagelkerke	0.959			0.828		

**Table 6:** FTIR-ATR spectroscopic analyses of the *n*-hexane (*n*-hex), ethyl acetate (EA), and ethanolic (EtOH) extracts of *Asparagopsis armata* (AA) and the aqueous extract (AQ) of *Sargassum muticum* (SM).

Extract	IR (cm^−1^)	Putative Signal Assignment	References
*n*-hex (AA)	2954	C–H stretching from CH and CH_2_ aliphatic groups	[29,30]
	2923	O–H stretching from carboxylic acid C–H stretching from CH_2_ aliphatic groups	[29,31]
	2853	C–H stretching from CH and CH_2_ aliphatic groups	[31,32]
	1710	C=O stretching from aliphatic ketone/carboxylic acid	[32,33]
	1650	C=O stretching from primary amide	[29]
	1537	C–N stretching from secondary amide	[29]
	1080	C–O stretching from primary alcohol	[30]
	720	C–Cl and C–Br stretching	[34,35]
AE (AA)	2954	C–H stretching from CH and CH_2_ aliphatic groups	[29,30]
	2923	O–H stretching from carboxylic acid C–H stretching from CH_2_ aliphatic groups	[29,31]
	2853	C–H stretching from CH and CH_2_ aliphatic groups	[31,32]
	1709	C=O stretching from aliphatic ketone/carboxylic acid	[32,33]
	1652	C=O stretching from primary amide	[29]
	1079	C–O stretching from ester	[33]
	720	C–Cl and C–Br stretching	[34,35]
EtOH (AA)	2924	O–H stretching from carboxylic acid	[29]
	2854	C–H stretching from alkane group	[32]
	1732	C=O stretching from aldehyde/ester	[36]
	1649	C=O stretching from primary amide	[29]
	1174	C–O stretching from ester	[37]
	1095	C–O stretching from carbohydrate	[32]
	1035	C–O stretching from carbohydrate (glycosidic bond)	[38]
AQ (SM)	2920	O–H stretching from carboxylic acid	[29]
	1603	C=O stretching from conjugated carbonyl	[30]
	1411	S=O stretching from sulfate	[38]
	1228	S=O asymmetric stretching from sulfate esters	[38]
	1027	C–O stretching from carbohydrate (glycosidic bond)	[38]

## Data Availability

Data will be provided upon reasonable request.

## Supplementary Materials

The following supporting information can be downloaded at: https://www.mdpi.com/article/10.3390/jof9020269/s1, Table S1: Mycelial growth inhibition rate (GIR) in mm/h obtained in the poisoned food technique assays by the extracts (*n*-hexane, ethyl acetate, aqueous, ethanolic, and hydroethanolic) of *Asparagopsis armata*, *Codium* sp., *Fucus vesiculosus,* and *Sargassum muticum* at 0.1, 0.5, and 1 mg/mL against *Alternaria alternata*, *Botrytis cinerea*, *Fusarium oxysporum,* and *Penicillium expansum*. The results obtained for the amphotericin B (growth inhibition control) at 30 µg/mL are also presented. The GIR is followed by the confidence interval [CI95], and, where possible, the inhibition percentage in comparison with the control growth rate (CGR) is indicated. Also, those conditions which do not follow a linear inhibition and do not adjust to the model are indicated as “n.s.” (non-significant), and those extracts that led to a higher growth rate are illustrated as “g.s.” (growth stimulant); Figure S1: Representative examples of the spore germination inhibition of *Botrytis cinerea* by the *n*-hexane, ethyl acetate, and ethanolic extracts of *Asparagopsis armata*. A and B are the MIC × 2 results: A.1: DMSO control (1 mg/mL); A.2: amphotericin B control (2 µg/mL); B.1: ethyl acetate (1 mg/mL); and B.2: ethanol (1 mg/mL). The letters a and b are the MFC and MFC x 2 results: a.1: DMSO control (0.5 mg/mL); a.2: DMSO control (1 mg/mL); a.3: amphotericin B control (2 µg/mL); b.1: *n*-hexane (1 mg/mL); b.2: ethyl acetate (1 mg/mL); b.3: ethanol (0.5 mg/mL); and b.4: ethanol (1 mg/mL); Figure S2: Representative examples of the spore germination inhibition of *Fusarium oxysporum* by the *n*-hexane, ethyl acetate, and ethanolic extracts of *Asparagopsis armata*. A and B are the MIC x 2 results: A.1: DMSO control (1 mg/mL); A.2: amphotericin B control (2 µg/mL); B.1: *n*-hexane (1 mg/mL); and B.2: ethyl acetate (1 mg/mL). The letters a and b are the MFC and MFC x 2 results, following the same correspondence of codes described for the MICs; Figure S3: FTIR-ATR spectroscopy spectra of the ethyl acetate (a), ethanolic (b), *n*-hexane (c), and extracts of *Asparagopsis armata* (AA) and the aqueous extract (AQ) of *Sargassum muticum* (d); Table S2: Estimate model table for the relationship among decay halo growth, time, and treatments with the seaweed aqueous extracts against *Botrytis cinerea*. The ratio at which the curves vary depending on the treatments used and the influence of time (Estimate) are represented, followed by the standard error (Std. Error), t and *p* values, the estimates with the influence of the control (Value), the same value without the logarithm (Linear value), and the percentage of the stimulant (positive) or inhibitory (negative) effect on the fruit decay in comparison with the control. Negative percentages represent decay stimulation. Furthermore, significant differences are indicated with asterisks, where * *p* < 0.05, ** *p* < 0.01, and *** *p* < 0.001; Table S3: Estimate model table for the relationship among decay halo growth, time, and treatments with the seaweed aqueous extracts against *Fusarium oxysporum*. The ratio at which the curves vary depending on the treatments used and the influence of time (Estimate) are represented, followed by the standard error (Std. Error), the t and *p* values, the estimates with the influence of the control (Value), the same value without the logarithm (Linear value), and the percentage of the stimulant (positive) or inhibitory (negative) effect on the fruit decay in comparison with the control. Negative percentages represent decay stimulation. Furthermore, significant differences are indicated with asterisks, where * *p* < 0.05, ** *p* < 0.01, and *** *p* < 0.001.
