# Supplementary material for: Seaweed Extracts to Control Postharvest Phytopathogenic Fungi in Rocha Pear
Source: J Fungi (Basel). 2023 Feb 17;9(2):269. doi: 10.3390/jof9020269 (PMC9967800; doi:10.3390/jof9020269)
Supplement: Supplementary file 1 [file jof-09-00269-s001.zip › jof-2200887-supplementary.pdf]

Supplementary Materials Content

Page 2 - Table S1: Mycelial growth inhibition rate (GIR) in mm/h obtained in the Poisoned food technique assays by the extracts (n-hexane, ethyl acetate, aqueous, ethanolic and hidroethanolic) of Asparagopsis armata, Codium sp., Fucus vesiculosus and Sargassum muticum at 0.1, 0.5 and 1 mg/mL against Alternaria alternata, Botrytis cinerea, Fusarium oxysporum and Penicillium expansum. The result obtained for the amphotericin B (growth inhibition control) at 30 μg/mL are also presented. The GIR followed by the confidence range [CI95] and where possible, the inhibition percentage in comparison with the control growth rate (CGR), are indicated. Also, those conditions which don't follow a linear inhibition and don't adjust to the model are indicated as "n.s." (non-significant) and those extracts that led to a higher growth rate are illustrated as "g.s." (growth stimulant);

Page 12 - Figure S1: Representative examples of the spore germination inhibition of Botrytis cinerea by the n-hexane, ethyl acetate and ethanolic extracts of Asparagopsis armata. A and B are the MIC x 2 results, A.1: DMSO control (1 mg/mL); A.2: amphotericin B control (2 μg/mL), B.1: ethyl acetate (1 mg/mL) and B.2: ethanol (1 mg/mL). The letters a and b are the MFC and MFC x 2 results, a.1: DMSO control (0.5 mg/mL); a.2: DMSO control (1 mg/mL); a.3: amphotericin B control (2 μg/mL), b.1: n-hexane (1 mg/mL); b.2: ethyl acetate (1 mg/mL); b.3: ethanol (0.5 mg/mL) and b.4: ethanol (1 mg/mL);

Page 12 - Figure S2: Representative examples of the spore germination inhibition of Fusarium oxysporum by the n-hexane, ethyl acetate and ethanolic extracts of Asparagopsis armata. A and B are the MIC x 2 results, A.1: DMSO control (1 mg/mL); A.2: amphotericin B control (2 μg/mL), B.1: n-hexane (1 mg/mL) and B.2: ethyl acetate (1 mg/mL). The letters a and b are the MFC and MFC x 2 results, following the same correspondence of codes described for the MICs;

Page 13 - Figure S3: FTIR-ATR spectroscopy spectra of the ethyl acetate (a), ethanolic (b), and n-hexane (c) extracts of Asparagopsis armata (AA) and the aqueous extract (AQ) of Sargassum muticum (d);

Page 13 - Table S2: Estimates model table for the relationship between decay halo's growth, time and treatments with the seaweeds' aqueous extracts against Botrytis cinerea. The ratio at which the curves vary depending on the treatments used and the influence of time (Estimate) are represented, followed by the standard error (Std. Error), t and p values, the estimates with the influence of the control (Value), the same value without the logarithm (Linear value) and the percentage of the stimulant (positive) or inhibitory (negative) effect on the fruit decay in comparison with the control. Negative percentages represent decay stimulation. Furthermore, significant differences are indicated with asterisks, where * $p < 0.05$, ** $p < 0.01$ and *** $p < 0.001$;

Page 14 - Table S3: Estimates model table for the relationship between decay halo's growth, time and treatments with the seaweeds' aqueous ex-tracts against Fusarium oxysporum. The ratio at which the curves vary depending on the treatments used and the influence of time (Estimate) are represented, followed by the standard error (Std. Error), t and p values, the estimates with the influence of the control (Value), the same value without the logarithm (Linear value) and the percentage of the stimulant (positive) or inhibitory (negative) effect on the fruit decay in comparison with the control. Negative percentages represent decay stimulation. Furthermore, significant differences are indicated with asterisks, where * $p < 0.05$, ** $p < 0.01$ and *** $p < 0.001$.

Table S1. Mycelial growth inhibition rate (GIR) in mm/h obtained in the Poisoned food technique assays by the extracts (*n*-hexane, ethyl acetate, aqueous, ethanolic and hidroethanolic) of *Asparagopsis armata*, *Codium* sp., *Fucus vesiculosus* and *Sargassum muticum* at 0.1, 0.5 and 1 mg/mL against *Alternaria alternata*, *Botrytis cinerea*, *Fusarium oxysporum* and *Penicillium expansum*. The result obtained for the amphotericin B (growth inhibition control) at 30 μg/mL are also presented. The GIR followed by the confidence range [CI95] and where possible, the inhibition percentage in comparison with the control growth rate (CGR), are indicated. Also, those conditions which don't follow a linear inhibition and don't adjust to the model are indicated as "n.s." (non-significant) and those extracts that led to a higher growth rate are illustrated as "g.s." (growth stimulant).

Mycelial growth inhibition rate							
Fungi	**Seaweed**	**GCR (mm/h)**	**Amphotericin B (30 μg/mL)**	**Extract**	**Concentration (mg/mL)**	**GIR (mm/h) (CI95)**	**Inhibition (%)**
A. alternata	*A. armata*	0.1351 (0.1274, 0.1428)	0.1181 (0.1131, 0.1232)	*n*-hexane	0.1	-0.0413 (-0.0496, -0.0330)	g.s.
					0.5	-0.0478 (-0.0509, -0.0447)	g.s.
					1	-0.0065 (-0.0100, -0.0029)	g.s.
				Ethyl acetate	0.1	-0.0202 (-0.0254, -0.0151)	g.s.
					0.5	-0.0235 (-0.0273, -0.0197)	g.s.
					1	-0.0164 (-0.0211, -0.0117)	g.s.
				EtOH	0.1	-0.0131 (-0.0182, -0.0081)	g.s.
					0.5	-0.0676 (-0.0751, -0.0601)	g.s.
					1	-0.0492 (-0.0536, -0.0447)	g.s.
				EtOH:H_2O	0.1	-0.0298 (-0.0331, -0.0264)	g.s.
					0.5	-0.0592 (-0.0639, -0.0546)	g.s.
					1	-0.0525 (-0.0582, -0.0468)	g.s.
				H_2O	0.1	-0.0090 (-0.0168, -0.0012)	g.s.
					0.5	-0.0370 (-0.0421, -0.0320)	g.s.
					1	-0.0465 (-0.0536, -0.0394)	g.s.
	Codium sp.	0.1351 (0.1274, 0.1428)	0.1181 (0.1131, 0.1232)	*n*-hexane	0.1	-0.0250 (-0.0343, -0.0157)	g.s.
					0.5	-0.0448 (-0.0519, -0.0377)	g.s.
					1	-0.0280 (-0.0317, -0.0244)	g.s.
				Ethyl acetate	0.1	-0.0090 (-0.0135, -0.0046)	g.s.
					0.5	-0.0220 (-0.0281, -0.0158)	g.s.

Fungi	Seaweed	GCR (mm/h)	Amphotericin B (30 µg/mL)	Extract	Concentration (mg/mL)	GIR (mm/h) (CI95)	Inhibition (%)
A. alternata	*Codium* sp.	0.1351 (0.1274, 0.1428)	0.1181 (0.1131, 0.1232)	Ethyl acetate	1	0.0066 (-0.0017, 0.0149)	n.s.
				EtOH	0.1	-0.0093 (-0.0138, -0.0048)	g.s.
					0.5	-0.0604 (-0.0686, -0.0522)	g.s.
					1	-0.0216 (-0.0349, -0.0083)	g.s.
				EtOH:H_2O	0.1	-0.0161 (-0.0215, -0.0108)	g.s.
					0.5	-0.0361 (-0.0442, -0.0280)	g.s.
					1	-0.0354 (-0.0410, -0.0299)	g.s.
				H_2O	0.1	0.0389 (0.0151, 0.0627)	29.3665
					0.5	0.0216 (-0.0067, 0.0499)	n.s.
					1	-0.0091 (-0.0148, -0.0033)	g.s.
	F. vesiculosus	0.1006 (0.0967, 0.1045)	0.0888 (0.0860, 0.0915)	*n*-hexane	0.1	-0.0254 (-0.0312, -0.0196)	g.s.
					0.5	-0.0311 (-0.0401, -0.0220)	g.s.
					1	-0.0057 (-0.0141, 0.0026)	n.s.
				Ethyl acetate	0.1	0.0005 (-0.0062, 0.0071)	n.s.
					0.5	-0.0095 (-0.0183, -0.0007)	g.s.
					1	0.0070 (-0.0002, 0.0141)	n.s.
				EtOH	0.1	0.0051 (-0.0021, 0.0123)	n.s.
					0.5	-0.0123 (-0.0211, -0.0035)	g.s.
					1	-0.0191 (-0.0259, -0.0122)	g.s.
				EtOH:H_2O	0.1	0.0131 (0.0082, 0.0181)	13.0517
					0.5	-0.0291 (-0.0349, -0.0233)	g.s.
					1	-0.0237 (-0.0291, -0.0183)	g.s.
				H_2O	0.1	-0.0264 (-0.0329, -0.0199)	g.s.
					0.5	-0.0318 (-0.0388, -0.0247)	g.s.
					1	-0.0752 (-0.0831, -0.0674)	g.s.
	S. muticum	0.1006 (0.0967, 0.1045)	0.0888 (0.0860, 0.0915)	*n*-hexane	0.1	-0.0291 (-0.0368, -0.0215)	g.s.

Fungi	Seaweed	GCR (mm/h)	Amphotericin B (30 µg/mL)	Extract	Concentration (mg/mL)	GIR (mm/h) (CI95)	Inhibition (%)
A. alternata	*S. muticum*	0.1006 (0.0967, 0.1045)	0.0888 (0.0860, 0.0915)	*n*-hexane	0.5	-0.0528 (-0.0570, -0.0487)	g.s.
					1	-0.0135 (-0.0171, -0.0099)	g.s.
				Ethyl acetate	0.1	-0.0364 (-0.0428, -0.0210)	g.s.
					0.5	-0.0552 (-0.0595, -0.0509)	g.s.
					1	-0.0421 (-0.0467, -0.0374)	g.s.
				EtOH	0.1	-0.0404 (-0.0448, -0.0360)	g.s.
					0.5	-0.0636 (-0.0676, -0.0597)	g.s.
					1	-0.0408 (-0.0440, -0.0376)	g.s.
				EtOH:H_2O	0.1	0.0258 (0.0227, 0.0289)	25.6362
					0.5	-0.0225 (-0.0272, -0.0179)	g.s.
					1	-0.0073 (-0.0103, -0.0043)	g.s.
				H_2O	0.1	-0.0463 (-0.0544, -0.0381)	g.s.
					0.5	-0.0617 (-0.0682, -0.0551)	g.s.
					1	-0.0639 (-0.0699, -0.0578)	g.s.
B. cinerea	*A. armata*	0.2181 (0.2040, 0.2321)	0.1417 (0.1345, 0.1488)	*n*-hexane	0.1	0.0066 (-0.0093, 0.0224)	3.0073
					0.5	0.0833 (0.0691, 0.0976)	38.2072
					1	0.1206 (0.1014, 0.1398)	55.2957
				Ethyl acetate	0.1	0.0123 (-0.0004, 0.0250)	n.s.
					0.5	0.0762 (0.0526, 0.0997)	34.9198
					1	0.1516 (0.1425, 0.1607)	69.5094
				EtOH	0.1	0.0613 (0.0454, 0.0772)	28.1247
					0.5	0.1118 (0.0987, 0.1249)	51.2609
					1	0.1109 (0.0898, 0.1319)	50.8482
				EtOH:H_2O	0.1	0.0424 (0.0308, 0.0540)	19.4223
					0.5	0.0720 (0.0437, 0.1003)	33.0078
					1	0.1475 (0.1400, 0.1549)	67.6295

Fungi	Seaweed	GCR (mm/h)	Amphotericin B (30 µg/mL)	Extract	Concentration (mg/mL)	GIR (mm/h) (CI95)	Inhibition (%)
B. cinerea	*A. armata*	0.2181 (0.2040, 0.2321)	0.1417 (0.1345, 0.1488)	H_2O	0.1	-0.0287 (-0.0347, -0.0227)	g.s.
					0.5	-0.0357 (-0.0473, -0.0241)	g.s.
					1	-0.0394 (-0.0490, -0.0297)	g.s.
	Codium sp.	0.2181 (0.2040, 0.2321)	0.1417 (0.1345, 0.1488)	*n*-hexane	0.1	-0.0238 (-0.0373, -0.0102)	g.s.
					0.5	0.0030 (-0.0063, 0.0123)	n.s.
					1	0.0289 (0.0234, 0.0344)	13.2691
				Ethyl acetate	0.1	0.0005 (-0.0110, 0.0119)	n.s.
					0.5	0.0245 (0.0107, 0.0384)	11.2517
					1	0.0269 (0.0175, 0.0362)	12.3109
				EtOH	0.1	-0.0389 (-0.0608, -0.0170)	g.s.
					0.5	-0.0324 (-0.0455, -0.0193)	g.s.
					1	-0.0299 (-0.0388, -0.0210)	g.s.
				EtOH:H_2O	0.1	0.0095 (-0.0016, 0.0206)	g.s.
					0.5	-0.0056 (-0.0128, 0.0017)	g.s.
					1	0.0107 (-0.0027, 0.0240)	g.s.
				H_2O	0.1	-0.0234 (-0.0340, -0.0128)	n.s.
					0.5	-0.0482 (-0.0630, -0.0333)	n.s.
					1	-0.0579 (-0.0802, -0.0356)	n.s.
	F. vesiculosus	0.2294 (0.2201, 0.2387)	0.1711 (0.1587, 0.1834)	*n*-hexane	0.1	0.0130 (-0.0066, 0.0325)	n.s.
					0.5	0.0644 (0.0590, 0.0697)	28.0514
					1	0.0977 (0.0859, 0.1095)	42.5850
				Ethyl acetate	0.1	0.0199 (0.0116, 0.0282)	8.6792
					0.5	0.0317 (0.0209, 0.0426)	13.8230
					1	0.0384 (0.0306, 0.0462)	16.7524
				EtOH	0.1	-0.0148 (-0.0222, -0.0074)	g.s.
					0.5	-0.0032 (-0.0110, 0.0045)	n.s.

Fungi	Seaweed	GCR (mm/h)	Amphotericin B (30 μg/mL)	Extract	Concentration (mg/mL)	GIR (mm/h) (CI95)	Inhibition (%)
B. cinerea	*F. vesiculosus*	0.2294 (0.2201, 0.2387)	0.1711 (0.1587, 0.1834)	EtOH	1	0.0009 (-0.0061, 0.0079)	n.s.
				EtOH:H_2O	0.1	0.0183 (0.0072, 0.0294)	7.9730
					0.5	0.0102 (0.0049, 0.0155)	4.4420
					1	0.0037 (-0.0008, 0.0082)	n.s.
				H_2O	0.1	0.1380 (0.1167, 0.1592)	60.1569
					0.5	0.0993 (0.0728, 0.1258)	43.2912
					1	0.0644 (0.0394, 0.0893)	28.0514
	S. muticum	0.2181 (0.2040, 0.2321)	0.1417 (0.1345, 0.1488)	*n*-hexane	0.1	0.0127 (-0.0095, 0.0350)	n.s.
					0.5	0.0130 (-0.0058, 0.0317)	n.s.
					1	0.0625 (0.0516, 0.0734)	27.2450
		0.2294 (0.2201, 0.2387)	0.1711 (0.1587, 0.1834)	Ethyl acetate	0.1	-0.0097 (-0.0206, 0.0011)	n.s.
					0.5	0.0308 (0.0201, 0.0415)	13.4220
					1	0.0417 (0.0362, 0.0471)	18.1648
				EtOH	0.1	0.0380 (0.0273, 0.0486)	n.s.
					0.5	0.0488 (0.0377, 0.0600)	21.2903
					1	0.0255 (0.0176, 0.0333)	11.0985
				EtOH:H_2O	0.1	0.0030 (-0.0112, 0.0172)	n.s.
					0.5	-0.0056 (-0.0114, 0.0003)	n.s.
					1	-0.0271 (-0.0406, -0.0136)	g.s.
				H_2O	0.1	-0.0058 (-0.0218, 0.0102)	g.s.
					0.5	-0.0206 (-0.0312, -0.0010)	g.s.
					1	-0.0213 (-0.0335, -0.0091)	g.s.
F. oxysporum	*A. armata*	0.1870 (0.1772, 0.1967)	0.1412 (0.1277, 0.1547)	*n*-hexane	0.1	0.0330 (0.0220, 0.0441)	17.6684
					0.5	0.0435 (0.0291, 0.0579)	23.2620
					1	0.0733 (0.0637, 0.0829)	39.1765
				Ethyl acetate	0.1	0.0243 (0.0158, 0.0329)	13.0160

Fungi	Seaweed	GCR (mm/h)	Amphotericin B (30 µg/mL)	Extract	Concentration (mg/mL)	GIR (mm/h) (CI95)	Inhibition (%)
F. oxysporum	*A. armata*	0.1870 (0.1772, 0.1967)	0.1412 (0.1277, 0.1547)	Ethyl acetate	0.5	0.0733 (0.0628, 0.0838)	39.1765
					1	0.0696 (0.0564, 0.0828)	37.2193
				EtOH	0.1	0.0037 (-0.0026, 0.0099)	n.s.
					0.5	0.0163 (0.0104, 0.0223)	8.7326
					1	0.0150 (0.0056, 0.0243)	8.0000
				EtOH:H_2O	0.1	0.0106 (0.0032, 0.0181)	5.6738
					0.5	0.0282 (0.0195, 0.0369)	15.0588
					1	0.0169 (0.0061, 0.0277)	9.0214
				H_2O	0.1	0.0098 (0.0054, 0.0142)	5.2642
					0.5	0.0053 (0.0004, 0.0106)	2.8567
					1	-0.0002 (-0.0055, 0.0052)	n.s.
F. oxysporum	*Codium* sp.	0.187 (0.1772, 0.1967)	0.1412 (0.1277, 0.1547)	*n*-hexane	0.1	-0.0106 (-0.0190, -0.0022)	g.s.
					0.5	0.0098 (0.0027, 0.0170)	4.8995
					1	0.0139 (0.0039, 0.0239)	6.9104
				Ethyl acetate	0.1	-0.0033 (-0.0109, 0.0043)	n.s.
					0.5	0.0181 (0.0111, 0.0250)	8.9851
					1	0.0217 (0.0129, 0.0305)	10.8060
				EtOH	0.1	-0.0073 (-0.0164, 0.0017)	g.s.
					0.5	0.0130 (0.0093, 0.0167)	g.s.
					1	0.0095 (0.0022, 0.0168)	g.s.
				EtOH:H_2O	0.1	-0.0018 (-0.0100, 0.0065)	n.s.
					0.5	0.0093 (0.0035, 0.0152)	6.4726
					1	0.0037 (-0.0053, 0.0126)	4.7114
				H_2O	0.1	-0.0086 (-0.0136, -0.0036)	n.s.
					0.5	-0.0165 (-0.0207, -0.0124)	4.6483
					1	-0.0157 (-0.0194, -0.0120)	n.s.

Fungi	Seaweed	GCR (mm/h)	Amphotericin B (30 µg/mL)	Extract	Concentration (mg/mL)	GIR (mm/h) (CI95)	Inhibition (%)
F. oxysporum	*F. vesiculosus*	0.1994 (0.1878, 0.2110)	0.1447 (0.1294, 0.1600)	*n*-hexane	0.1	0.0037 (-0.0021, 0.0096)	1.8626
					0.5	0.0390 (0.0316, 0.0464)	19.5587
					1	0.0253 (0.0170, 0.0336)	12.7081
				Ethyl acetate	0.1	0.0015 (-0.0021, 0.0050)	n.s.
					0.5	0.0015 (-0.0024, 0.0053)	n.s.
					1	0.0084 (0.0018, 0.0150)	4.2096
				EtOH	0.1	-0.0014 (-0.0051, 0.0024)	n.s.
					0.5	-0.0091 (-0.0127, -0.0054)	g.s.
					1	0.0079 (-0.0001, 0.0158)	n.s.
				EtOH:H_2O	0.1	-0.0018 (-0.0061, 0.0025)	n.s.
					0.5	0.0062 (-0.0007, 0.0130)	n.s.
					1	0.0018 (-0.0067, 0.0104)	n.s.
				H_2O	0.1	0.0212 (0.0163, 0.0262)	10.6369
					0.5	0.0169 (0.0125, 0.0213)	n.s.
					1	0.0082 (0.0041, 0.0122)	4.0948
	S. muticum	0.1994 (0.1878, 0.2110)	0.1447 (0.1294, 0.1600)	*n*-hexane	0.1	0.0094 (0.0044, 0.0145)	9.3807
					0.5	0.0079 (-0.0007, 0.0165)	n.s.
					1	0.0343 (0.0292, 0.0395)	34.1352
				Ethyl acetate	0.1	-0.0042 (-0.0068, -0.0016)	g.s.
					0.5	0.0149 (0.0080, 0.0218)	14.7913
					1	0.0079 (-0.0008, 0.0166)	n.s.
				EtOH	0.1	0.0105 (0.0053, 0.0158)	10.4672
					0.5	0.0147 (0.0087, 0.0206)	14.5626
					1	0.0089 (0.0021, 0.0156)	8.7992
				EtOH:H_2O	0.1	0.0024 (-0.0034, 0.0082)	n.s.
					0.5	0.0024 (-0.0019, 0.0068)	n.s.

Fungi	Seaweed	GCR (mm/h)	Amphotericin B (30 μg/mL)	Extract	Concentration (mg/mL)	GIR (mm/h) (CI95)	Inhibition (%)
F. oxysporum	*S. muticum*	0.1994 (0.1878, 0.2110)	0.1447 (0.1294, 0.1600)	$EtOH:H_2O$	1	-0.0018 (-0.0088, 0.0053)	n.s.
				H_2O	0.1	0.0129 (0.0075, 0.0183)	12.8231
					0.5	0.0137 (0.0078, 0.0197)	13.6581
					1	0.0082 (0.0020, 0.0144)	8.1928
P. expansum	*A. armata*	0.0610 (0.0602, 0.0618)	0.0445 (0.0437, 0.0456)	*n*-hexane	0.1	0.0031 (0.0009, 0.0054)	5.1197
					0.5	0.0055 (0.0037, 0.0073)	8.9951
					1	0.0082 (0.0056, 0.011)	13.4852
				Ethyl acetate	0.1	0.0051 (0.0030, 0.0073)	8.4311
					0.5	0.0146 (0.0122, 0.0170)	23.9836
					1	0.0272 (0.0235, 0.0309)	44.5902
				EtOH	0.1	-0.0006 (-0.0016, 0.0004)	n.s.
					0.5	0.0033 (0.0021, 0.0045)	5.4180
					1	0.0017 (0.0007, 0.0027)	2.7164
				$EtOH:H_2O$	0.1	0.0042 (0.0034, 0.0051)	6.9180
					0.5	0.0055 (0.0041, 0.0069)	9.0016
					1	0.0036 (0.0022, 0.0050)	5.8590
				H_2O	0.1	0.0018 (0.0011, 0.0025)	2.9607
					0.5	-0.0001 (-0.0009, 0.0006)	n.s.
					1	-0.0014 (-0.002, -0.0008)	g.s.
	Codium sp.	0.0616 (0.0608, 0.0624)	0.0452 (0.0442, 0.0462)	*n*-hexane	0.1	-0.0091 (-0.0104, -0.0079)	g.s.
					0.5	0.0027 (0.0004, 0.0050)	4.4017
					1	-0.0001 (-0.0011, 0.0010)	n.s.
				Ethyl acetate	0.1	0.0022 (0.0003, 0.004)	3.5834
					0.5	0.0036 (0.0019, 0.0053)	5.8370
					1	0.0106 (0.0079, 0.0132)	17.1456
				EtOH	0.1	0.0052 (0.0030, 0.0074)	8.3926

Fungi	Seaweed	GCR (mm/h)	Amphotericin B (30 μg/mL)	Extract	Concentration (mg/mL)	GIR (mm/h) (CI95)	Inhibition (%)
P. expansum	*Codium* sp.	0.0616 (0.0608, 0.0624)	0.0452 (0.0442, 0.0462)	EtOH	0.5	0.0143 (0.0133, 0.0152)	23.1369
P. expansum	*Codium* sp.	0.0616 (0.0608, 0.0624)	0.0452 (0.0442, 0.0462)	EtOH	1	0.0034 (0.0020, 0.0049)	5.5837
P. expansum	*Codium* sp.	0.0616 (0.0608, 0.0624)	0.0452 (0.0442, 0.0462)	EtOH:H_2O	0.1	0.0069 (0.0055, 0.0084)	11.2664
P. expansum	*Codium* sp.	0.0616 (0.0608, 0.0624)	0.0452 (0.0442, 0.0462)	EtOH:H_2O	0.5	0.0125 (0.0110, 0.0140)	20.2468
P. expansum	*Codium* sp.	0.0616 (0.0608, 0.0624)	0.0452 (0.0442, 0.0462)	EtOH:H_2O	1	0.0058 (0.0034, 0.0082)	9.3846
P. expansum	*Codium* sp.	0.0616 (0.0608, 0.0624)	0.0452 (0.0442, 0.0462)	H_2O	0.1	0.0016 (0.0005, 0.00265)	2.5183
P. expansum	*Codium* sp.	0.0616 (0.0608, 0.0624)	0.0452 (0.0442, 0.0462)	H_2O	0.5	0.0146 (0.01360, 0.0156)	23.7376
P. expansum	*Codium* sp.	0.0616 (0.0608, 0.0624)	0.0452 (0.0442, 0.0462)	H_2O	1	-0.0038 (-0.0047, -0.0029)	g.s.
P. expansum	*F. vesiculosus*	0.0100 (0.0966, 0.1034)	0.0786 (0.0761, 0.0811)	*n*-hexane	0.1	-0.0156 (-0.0175, -0.0137)	g.s.
P. expansum	*F. vesiculosus*	0.0100 (0.0966, 0.1034)	0.0786 (0.0761, 0.0811)	*n*-hexane	0.5	-0.0144 (-0.0161, -0.0127)	g.s.
P. expansum	*F. vesiculosus*	0.0100 (0.0966, 0.1034)	0.0786 (0.0761, 0.0811)	*n*-hexane	1	-0.0172 (-0.0194, -0.0150)	g.s.
P. expansum	*F. vesiculosus*	0.0100 (0.0966, 0.1034)	0.0786 (0.0761, 0.0811)	Ethyl acetate	0.1	-0.0074 (-0.0102, -0.0046)	g.s.
P. expansum	*F. vesiculosus*	0.0100 (0.0966, 0.1034)	0.0786 (0.0761, 0.0811)	Ethyl acetate	0.5	-0.0135 (-0.0158, -0.0113)	g.s.
P. expansum	*F. vesiculosus*	0.0100 (0.0966, 0.1034)	0.0786 (0.0761, 0.0811)	Ethyl acetate	1	-0.0169 (-0.0195, -0.0142)	g.s.
P. expansum	*F. vesiculosus*	0.0610 (0.0602, 0.0618)	0.0445 (0.0437, 0.0456)	EtOH	0.1	-0.0036 (-0.0043, -0.0028)	g.s.
P. expansum	*F. vesiculosus*	0.0610 (0.0602, 0.0618)	0.0445 (0.0437, 0.0456)	EtOH	0.5	-0.0027 (-0.0039, -0.0015)	g.s.
P. expansum	*F. vesiculosus*	0.0610 (0.0602, 0.0618)	0.0445 (0.0437, 0.0456)	EtOH	1	0.0018 (-0.0004, 0.0041)	n.s.
P. expansum	*F. vesiculosus*	0.0100 (0.0966, 0.1034)	0.0786 (0.0761, 0.0811)	EtOH:H_2O	0.1	-0.0143 (-0.0157, -0.0128)	g.s.
P. expansum	*F. vesiculosus*	0.0100 (0.0966, 0.1034)	0.0786 (0.0761, 0.0811)	EtOH:H_2O	0.5	-0.0209 (-0.0228, -0.0189)	g.s.
P. expansum	*F. vesiculosus*	0.0100 (0.0966, 0.1034)	0.0786 (0.0761, 0.0811)	EtOH:H_2O	1	-0.0189 (-0.0207, -0.0171)	g.s.
P. expansum	*F. vesiculosus*	0.0100 (0.0966, 0.1034)	0.0786 (0.0761, 0.0811)	H_2O	0.1	-0.0165 (-0.0179, -0.0152)	g.s.
P. expansum	*F. vesiculosus*	0.0100 (0.0966, 0.1034)	0.0786 (0.0761, 0.0811)	H_2O	0.5	-0.0140 (-0.0160, -0.0120)	g.s.
P. expansum	*F. vesiculosus*	0.0100 (0.0966, 0.1034)	0.0786 (0.0761, 0.0811)	H_2O	1	-0.0148 (-0.0163, -0.0134)	g.s.
P. expansum	*S. muticum*	0.0618 (0.0609, 0.0627)	0.0455 (0.0444, 0.0465)	*n*-hexane	0.1	0.0075 (0.0049, 0.0101)	9.3074
P. expansum	*S. muticum*	0.0618 (0.0609, 0.0627)	0.0455 (0.0444, 0.0465)	*n*-hexane	0.5	0.0092 (0.0071, 0.0114)	14.9126
P. expansum	*S. muticum*	0.0618 (0.0609, 0.0627)	0.0455 (0.0444, 0.0465)	*n*-hexane	1	0.0197 (0.0165, 0.0228)	31.8123

Fungi	Seaweed	GCR (mm/h)	Amphotericin B (30 μg/mL)	Extract	Concentration (mg/mL)	GIR (mm/h) (CI95)	Inhibition (%)
P. expansum	*S. muticum*	0.0618 (0.0609, 0.0627)	0.0455 (0.0444, 0.0465)	Ethyl acetate	0.1	-0.0079 (-0.0088, -0.0071)	g.s.
					0.5	-0.0064 (-0.0075, -0.0053)	g.s.
					1	-0.0058 (-0.0073, -0.0044)	g.s.
		0.0809 (0.0744, 0.0874)	0.0623 (0.0605, 0.0642)	EtOH	0.1	-0.0160 (-0.0178, -0.0143)	g.s.
					0.5	-0.0203 (-0.0227, -0.0179)	g.s.
					1	-0.0156 (-0.0185, -0.0126)	g.s.
				EtOH:H_2O	0.1	-0.0154 (-0.0175, -0.0134)	g.s.
					0.5	-0.0225 (-0.0251, -0.0198)	g.s.
					1	-0.0271 (-0.0298, -0.0245)	g.s.
				H_2O	0.1	-0.0153 (-0.0167, -0.0139)	g.s.
					0.5	-0.0136 (-0.0148, -0.0124)	g.s.
					1	-0.0165 (-0.0179, -0.0151)	g.s.

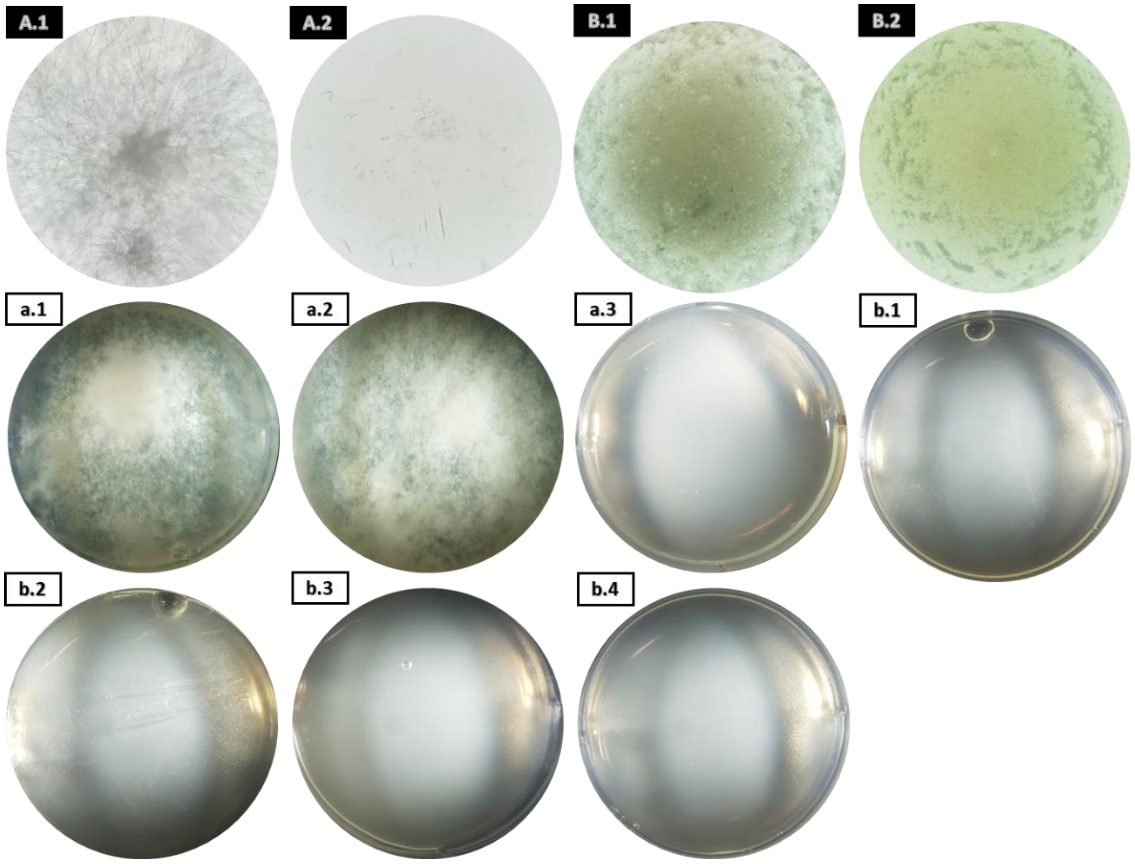

Figure S1. Representative examples of the spore germination inhibition of *Botrytis cinerea* by the *n*-hexane, ethyl acetate and ethanolic extracts of *Asparagopsis armata*. A and B are the MIC x 2 results, A.1: DMSO control (1 mg/mL); A.2: amphotericin B control (2 µg/mL), B.1: ethyl acetate (1 mg/mL) and B.2: ethanol (1 mg/mL). The letters a and b are the MFC and MFC x 2 results, a.1: DMSO control (0.5 mg/mL); a.2: DMSO control (1 mg/mL); a.3: amphotericin B control (2 µg/mL), b.1: *n*-hexane (1 mg/mL); b.2: ethyl acetate (1 mg/mL); b.3: ethanol (0.5 mg/mL) and b.4: ethanol (1 mg/mL).

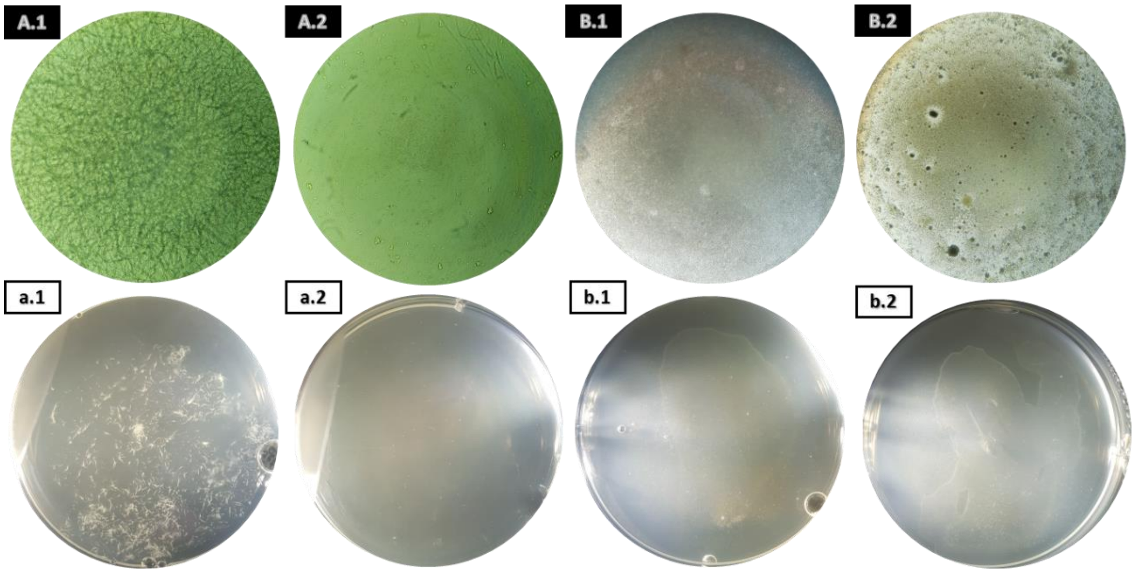

Figure S2. Representative examples of the spore germination inhibition of *Fusarium oxysporum* by the *n*-hexane, ethyl acetate and ethanolic extracts of *Asparagopsis armata*. A and B are the MIC x 2 results, A.1: DMSO control (1 mg/mL); A.2: amphotericin B control (2 µg/mL), B.1: *n*-hexane (1 mg/mL) and B.2: ethyl acetate (1 mg/mL). The letters a and b are the MFC and MFC x 2 results, following the same correspondence of codes described for the MICs.

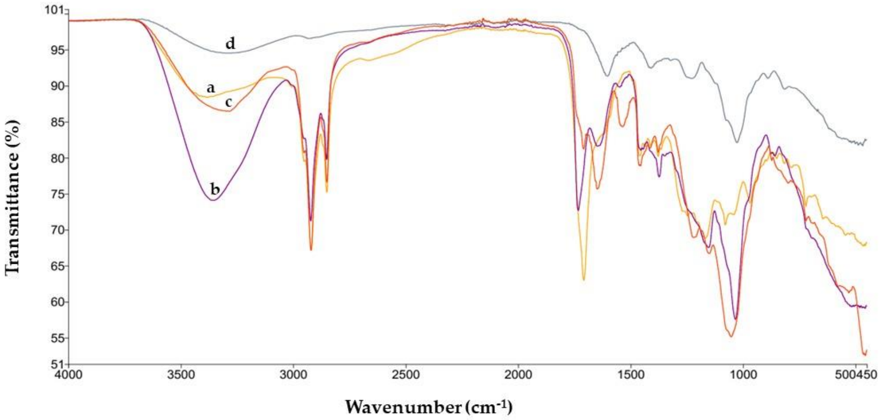

Figure S3. FTIR-ATR spectroscopy spectra of the ethyl acetate (a), ethanolic (b), and *n*-hexane (c) extracts of *Asparagopsis armata* (AA) and the aqueous extract (AQ) of *Sargassum muticum* (d).

Table S2. Estimates table of the linear general model for the relationship between decay halo's growth, time and treatments with the seaweeds' aqueous extracts against *Botrytis cinerea*. The ratio at which the curves vary depending on the treatments used and the influence of time (Estimate) are represented, followed by the standard error (Std. Error), t and p values, the estimates with the influence of the control (Value), the same value without the logarithm (Linear value) and the percentage of the stimulant (positive) or inhibitory (negative) effect on the fruit decay in comparison with the control. Negative percentages represent decay stimulation. Furthermore, significant differences are indicated with asterisks, where * $p < 0.05$, ** $p < 0.01$ and *** $p < 0.001$.

	Estimate	Std. Error	t value	p value	Value	Linear value	Real effect (%)
Control	-3.077	0.236	-13.05	0.000	-3.077	0.046	0.000
Asparagopsis armata	0.481	0.121	3.971	0.000	-2.596	0.075	61.799***
Codium sp.	0.030	0.134	0.222	0.824	-3.047	0.047	3.012
Fucus vesiculosus	-0.373	0.149	-2.503	0.013	-3.450	0.032	-31.145*
Sargassum muticum	-0.849	0.174	-4.883	0.000	-3.926	0.020	-57.213***
Time (h)	0.020	0.001	20.944	0.000	-3.057	0.047	2.012***
Observations	658						
R^2 Nagelkerke	0.959						

Table S3. Estimates table of the linear general model for the relationship between decay halo's growth, time and treatments with the seaweeds' aqueous extracts against *Fusarium oxysporum*. The ratio at which the curves vary depending on the treatments used and the influence of time (Estimate) are represented, followed by the standard error (Std. Error), t and p values, the estimates with the influence of the control (Value), the same value without the logarithm (Linear value) and the percentage of the stimulant (positive) or inhibitory (negative) effect on the fruit decay in comparison with the control. Negative percentages represent decay stimulation. Furthermore, significant differences are indicated with asterisks, where * $p < 0.05$, ** $p < 0.01$ and *** $p < 0.001$.

	Estimate	Std. Error	t value	p value	Value	Linear value	Real effect (%)
Control	-1.055	0.131	-8.024	0.000	-1.055	0.348	0.000
Asparagopsis armata	0.289	0.098	2.948	0.003	-0.766	0.465	33.497**
Codium sp.	-0.456	0.118	-3.847	0.000	-1.511	0.221	-36.603***
Fucus vesiculosus	0.012	0.104	0.114	0.909	-1.043	0.352	1.196
Sargassum muticum	0.025	0.104	0.237	0.813	-1.030	0.357	2.502
Time (h)	0.017	0.001	20.649	0.000	-1.038	0.354	1.732***
Observations	418						
R^2 Nagelkerke	0.828						
